# Influenza-Induced CD103^+^ T Resident Memory Cells Exhibit Enhanced Functional Avidity over CD103^−^ Memory T Cells in the Mediastinal Lymph Node

**DOI:** 10.4049/immunohorizons.2100074

**Published:** 2022-10-11

**Authors:** Sequoia D. Crooks, Steven M. Varga, John T. Harty

**Affiliations:** * Interdisciplinary Graduate Program in Immunology, Carver College of Medicine, University of Iowa, Iowa City, IA; † Department of Microbiology and Immunology, Carver College of Medicine, University of Iowa, Iowa City, IA; ‡ Department of Pathology, Carver College of Medicine, University of Iowa, Iowa City, IA

## Abstract

Influenza virus–specific tissue-resident memory CD8 T cells (Trms) targeting conserved viral proteins provide strain-transcending heterosubtypic immunity to infection. Trms in the lung combat reinfection through rapid cytolytic function and production of inflammatory cytokines to recruit other immune cells. Influenza-specific Trms are also generated in the lung draining mediastinal lymph node (mLN) and can provide immunity to heterologous virus infection in this tissue, although their role in combating influenza infection is less well defined. Functional avidity, a measure of T cell sensitivity to Ag stimulation, correlates with control of viral infection and may be important for immune detection of recently infected cells, when low numbers of surface peptide–MHC complexes are displayed. However, the functional avidity of influenza-specific Trms has not been previously compared with that of other memory CD8 T cell subsets. In this article, a methodology is presented to compare the functional avidity of CD8 T cell subsets across murine tissues, with a focus on influenza-specific mLNs compared with splenic CD8 T cells, by stimulating both populations in the same well to account for CD8 T cell–extrinsic variables. The functional avidity of influenza-specific mLN effector CD8 T cells is slightly increased relative to splenic effector CD8 T cells. However, CD103^+^ mLN Trms display increased functional avidity compared with splenic memory CD8 T cells and CD103^−^ memory CD8 T cells within the mLN. In contrast, lung-derived CD103^+^ Trms did not exhibit enhanced functional avidity. mLN CD103^+^ Trms also exhibit increased TCR expression, providing a potential mechanism for their enhanced functional avidity.

## INTRODUCTION

Despite the availability of seasonal vaccines, influenza remains a major health and economic burden. Respiratory disease caused by seasonal influenza is responsible for an estimated annual 20,000–30,000 deaths in the United States and 290,000–650,000 deaths worldwide (1, 2). Pandemic strains of influenza A virus (IAV) may cause increased mortality, particularly among young adults; this occurred with the 1918 Spanish flu, which is estimated to have caused >50 million deaths (3).

One of the reasons that influenza viruses present an ongoing health threat despite prior exposures and vaccination strategies is due to antigenic variation in the surface molecules hemagglutinin (HA) and neuraminidase (NA), which can occur through antigenic drift over time and sudden antigenic shifts (4). Because current immunization strategies primarily aim to generate protective Ab responses against HA/NA molecules, circulation of strains that escape a given year’s vaccine presents an ongoing challenge. Therefore, development of a vaccine that produces strain-transcending (heterosubtypic) immunity is of key interest (5, 6).

One potential avenue to heterosubtypic immunity can be found in CD8 T cells, which play a well-established role in the clearance of primary influenza virus infection, and importantly can react to epitopes that are conserved across influenza strains (such as those derived from the nucleoprotein) to provide heterosubtypic immunity (7, 8). During the initial response to infection, Ag-specific effector CD8 T cells localize to the lung tissue and lung draining mediastinal lymph node (mLN) (9, 10). The peak of the influenza-specific CD8 T cell response in the lungs after experimental infection of mice occurs around 10 d postinfection (dpi), coinciding with viral clearance (11). Effector CD8 T cells responding to influenza exhibit multiple antiviral mechanisms, including expression of cytolytic molecules, cytokines, and chemokines, and adoptive transfer of influenza-specific effector CD8 T cells reduces viral load, loss of lung function, and mortality (12).

After the resolution of infection, influenza-specific memory CD8 T cells persist systemically and within the lungs, where they can be identified for up to 2 y postinfection in mice (13). This population is key in providing heterosubtypic immunity, and correspondingly CD8 T cell depletion reduces heterosubtypic immunity against IAV strain X31 (H3N2) in mice previously immunized by infection with IAV strain PR8 (H1N1) (14). One particularly important subset of the memory population is Ag-specific and nonrecirculating CD69^+^103^+^ tissue-resident memory CD8 T cell (Trm) populations, which are generated in the lung and lung draining mLNs after IAV infection (10). The numbers of lung Trms correspond with heterosubtypic immunity, and intratracheal transfer of Trm is sufficient to reduce viral load on rechallenge (15-17). Protective Trm populations can be generated through infection or local vaccination; for example, both intranasal (i.n.) administration of a live attenuated influenza vaccine and Ab-mediated targeting of Ag to respiratory dendritic cells (DCs) generated Trm and heterosubtypic protection in mice (17, 18). Trm may thus be an attractive target in producing heterosubtypic immunity against influenza (5).

Given the role of Trms in providing protective heterosubtypic immunity against influenza, we hypothesized they might display increased Ag sensitivity to enable their rapid activation on reinfection. One measure of Ag sensitivity has been termed functional avidity (19). Functional avidity is defined by the ability of a T cell to respond to different concentrations of cognate Ag, and it can be described quantitatively through the EC_50_ value, or calculated concentration of peptide at which half of the maximal frequency of T cells capable of producing cytokine at saturating Ag concentrations do so (20, 21). Functional avidity has been correlated with the control of viral infections in humans and in murine models (22-26). Little is known of how functional avidity is regulated in Trm populations specifically; Trm subsets may exhibit an enrichment of high-affinity TCRs, but other factors, such as cytokine signals, membrane organization, and the level of TCR expression, can affect avidity independently of TCR affinity (19, 21, 27-31).

Although prior studies have focused on TCR affinity for Ag (27, 32), we are primarily interested in how functional avidity may be regulated within Trm populations independently of this characteristic. Although responding quickly on reinfection is a key function of Trms, it remains unknown whether differences in TCR clonotypes and environmental signals integrate to produce functional differences in the ability of Trms to produce cytokine in response to low levels of Ag. In this study, we investigate functional avidity in splenic, lung, and mLN CD8 T cell populations generated after influenza virus infection, at both effector and memory time points, and identify TCR expression as a potential mechanism underlying the regulation of functional avidity in these subsets.

## MATERIALS AND METHODS

### Mice

Female Thy1.2 C57BL/6 (B6) mice were purchased from Charles River and infected or immunized at 8–12 wk of age. Thy1.1/1.1 homozygous P14 mice expressing a transgenic (Tg) TCR specific to the gp33–41 epitope of lymphocytic choriomeningitis virus (LCMV), on a C57BL/6 background, were obtained from the laboratory of M. Bevan (University of Washington) and bred in-house. All experimental procedures using mice were approved by the University of Iowa’s Animal Care and Use Committee, under U.S. Public Health Service assurance, Office of Laboratory Animal Welfare guidelines.

### Adoptive transfer

Peripheral blood cells from naive Thy1.1/1.1 P14 TCR-Tg mice were isolated, and RBCs were lysed with 1× Vitalyse (CMDG), washed, and characterized by flow cytometry for the frequency of CD8^+^ Thy1.1^+^ Vα2^+^ P14 TCR-Tg T cells. Cells were resuspended in 1× PBS at a concentration of 1.25 × 10^5^ P14 cells/ml, and 200 μl (the equivalent of 2.5 × 10^4^ P14 cells) was transferred via i.v. injection into each naive Thy1.2/1.2 recipient.

### Virus and infection

Recombinant influenza A/PR/8/34 (H1N1) expressing gp33–41 (PR8-gp33) has been described previously (33) and was the gift of S. Varga (University of Iowa). After anesthetization with ketamine/xylazine injected i.p., mice were infected i.n. with 25 μl of virus diluted in 1× PBS (~10 PFUs) and monitored daily for weight loss. Mice losing ≥30% of body weight were euthanized per Institutional Animal Care and Use Committee protocol.

### DC prime

Naive female B6 mice were injected with 5 × 10^6^ B16-Flt3L cells (a gift of M. Bevan) i.p. 16 d later, and mice were injected with 1 μg LPS i.v. (Sigma). Spleens were harvested 15–17 h later and chopped into small pieces in collagenase (125 U/ml) and DNase (0.1 mg/ml), and incubated at 37°C while shaking at 100 rpm for 20 min. Subsequently, they were homogenized through a 70-μm cell strainer, and RBCs were lysed in 1× Vitalyse. Splenocytes were incubated in 2 parts RP10:1 part B16-Flt3L conditioned media supplemented with rGM-CSF (Becton Dickinson) (1000 U/ml) and 2 μM gp33–41 (catalog number [Cat]: AS-61296; AnaSpec) and shaken at 100 rpm for 2 h at 37°C. They were then washed and isolated using anti-CD11c microbeads (Order no.: 130-108-338; Miltenyi Biotec) and magnetic separation on LS columns, as described in the manufacturer’s protocol (Miltenyi Biotec). Isolated gp33-pulsed DCs were counted using a hemocytometer and resuspended at 2.5 × 10^6^ cells/ml in 1× PBS. Naive recipients were injected with 200 μl i.v. (the equivalent of 5 × 10^5^ DCs).

### Tissue preparation and stimulation

At the indicated time points, 0.25 μg anti-CD45 allophycocyanin (30-F11, Cat: 103112; BioLegend) in 100 μl 1× PBS was administered i.n. to IAV-immune mice 5 min before sacrifice, followed by i.v. injection of 2 μg anti–CD45.2-BV421 (104, Cat: 109832; BioLegend) in 200 μl of 1× PBS 2 min before euthanasia. mLNs, lungs, and spleens were harvested. mLNs were then processed by homogenization and resuspension in 600–750 μl RP10, depending on the volume required for a given experimental layout. Lungs were chopped into small pieces in collagenase (125 U/ml) and DNase (0.1 mg/ml) and incubated at 37°C while shaking at 100 rpm for 1 h. Subsequently, they were homogenized through a 70-μm cell strainer and separated on a gradient of 35% Percoll in HBSS at 1500 rpm at room temperature for 5 min.

Splenocytes were processed in the same manner as the tissue to which they were to be compared (either the same protocol as mLN or lung), then prelabeled through a 20-min incubation with anti-Thy1.1-PerCP (OX-7, Cat: 202528; BioLegend) (1:1000) in FACS buffer at 4°C, washed, and resuspended in the minimum volume needed for a given experiment (600–750 μl RP10). The concentration was then adjusted to 4 × 10^7^ cells/ml or, if <4 × 10^7^ cells/ml were recovered, resuspended at a concentration equal to that of the sample with the lowest number of splenocytes for consistency across samples. Using this methodology, final splenocyte numbers fell within the range of 1–2 × 10^6^ cells/well but were consistent within a given experiment. mLN or lung samples were combined with an equal volume of prelabeled splenocytes. A total of 100 μl of this cell mixture was added to wells of a 96-well plate, with one well for each titrated Ag concentration and one well as a no-peptide control. Titrated gp33 in the range of 2 × 10^−8^ to 1 × 10^−11^ M was generated through serial dilution of a 1 mM stock in RP10 with 1× brefeldin A (Cat: 420601; BioLegend). A total of 100 μl of each Ag concentration was added to each stimulated well, whereas the no-peptide control received RP10 1 brefeldin A alone.

Cells were stimulated at 37°C for 5 h, after which they were washed in 1× PBS and stained for surface markers using the following Abs at the indicated dilutions in 1× PBS: anti-CD8 BV785 (53-6.7, Cat: 100750; eBioscience) (1:200), anti-CD103 PE (2E7, Cat: 121406) (1:200), anti-Vα2 FITC (B20.1, Cat: 553288; BD), anti-CD11a BV510 (M17/4, Cat: 624144; BD) (1:300), anti-Thy1.1 Af700 (OX-7, Cat: 202516; BioLegend) (1:1000), and viability stain ef780 (Cat: 565388; BD) (1:1000). Cells were incubated for 20 min at 4°C, then washed and resuspended in 100 μl 1× permeabilization/fixation buffer for 10 min at 4°C (Cat: 51-2090KZ; BD). They were then washed in 1× permeabilization buffer (Cat: TNB-1213-L150; Tonbo Biosciences) and resuspended in anti–IFN-γ FITC (XMG1.2, Cat: 505806) (1:200) in 1× permeabilization buffer. Cells were incubated for 20 min at 4°C, then washed and resuspended in FACS buffer for analysis via flow cytometry. Flow cytometry data were acquired in FACS Diva using an LSR Fortessa (Becton Dickinson) and analyzed using FlowJo software (Version 10.7.0; Becton Dickinson).

### Statistical analysis

For the generation of functional avidity curves, the raw frequencies of IFN-γ-producing cells at each Ag concentration for a given biological replicate were exported to Microsoft Excel (Version 16.49 for Mac). Stimulation-induced cytokine production was calculated by subtraction of the background IFN-γ production in the no-peptide control from all Ag concentrations. Percent of maximum IFN-γ production was then calculated by dividing the frequency of IFN-γ^+^ cells at each Ag concentration by the frequency at the maximum Ag concentration, then multiplying by 100%. Negative values were corrected to 0%, and values >100% were corrected to 100%. These data were then imported to Prism 8 software (GraphPad). Individual Ag sensitivity curves were generated for each biological replicate using the Prism function “[Agonist] vs. normalized response,” and EC_50_ values were obtained. Grouped avidity curves were also generated to display the mean IFN-γ response and SD between biological replicates at each peptide dilution.

Statistical differences between two groups were evaluated using a paired, two-tailed *t* test, with pairing between cell populations obtained from the same mouse. Statistical differences between more than two groups were evaluated using a one-way ANOVA with Tukey’s multiple comparison test, with matching between cell populations obtained from the same mouse.

Statistical significance was assigned as *p < 0.05, **p < 0.01, ***p < 0.001, and ****p < 0.0001.

## RESULTS

### Comparison of functional avidity of T cells between tissues

Ag sensitivity of CD8 T cells can be assessed using direct ex vivo peptide stimulation and cytokine production. Multiple factors can affect peptide-induced cytokine production by a CD8 T cell population: CD8 T cell–intrinsic factors, the ability of tissue APCs to stimulate a T cell response, and competition between cells for interaction with APCs presenting cognate Ag (34). To assess T cell–intrinsic differences in functional avidity across tissues, we have established a methodology wherein splenocytes and tissue cells are stimulated within the same well, and the percent of each population producing IFN-γ was determined at multiple Ag concentrations. We have chosen to focus on the cytokine IFN-γ given that Trms, including those present in the airway, can provide protection from influenza in an IFN-γ–dependent manner (16).

Functional avidity is a measure of a T cell’s ability to produce cytokines in response to titrated Ag concentrations and is a function of both TCR affinity for cognate Ag and dynamic regulation (19, 31, 34, 35). To investigate how the functional avidity of CD8 T cell populations generated after IAV infection was regulated independently of the variable of TCR affinity, we made use of TCR-Tg P14 cells with a fixed TCR that is specific for the epitope gp33–41, derived from LCMV, and recombinant IAV expressing the gp33 epitope (PR8-gp33). To avoid initial competition for Ag stimulation and ensure all P14 cells received similar activation signals, we transferred a relatively low number of naive P14 cells (2.5 × 10^4^) and modified a prime-boost strategy (36) to generate sufficient effector and memory P14 cells to avoid pooling cells from different mice. Naive Thy1.1/1.1 P14 cells were transferred into naive Thy1.2/1.2 B6 mice that were primed the next day with gp33-pulsed mature DCs. One week later, mice were boosted by i.n. infection with PR8-gp33, yielding sufficient influenza-experienced memory P14 numbers in the lung and mLN.

**FIGURE 1.**
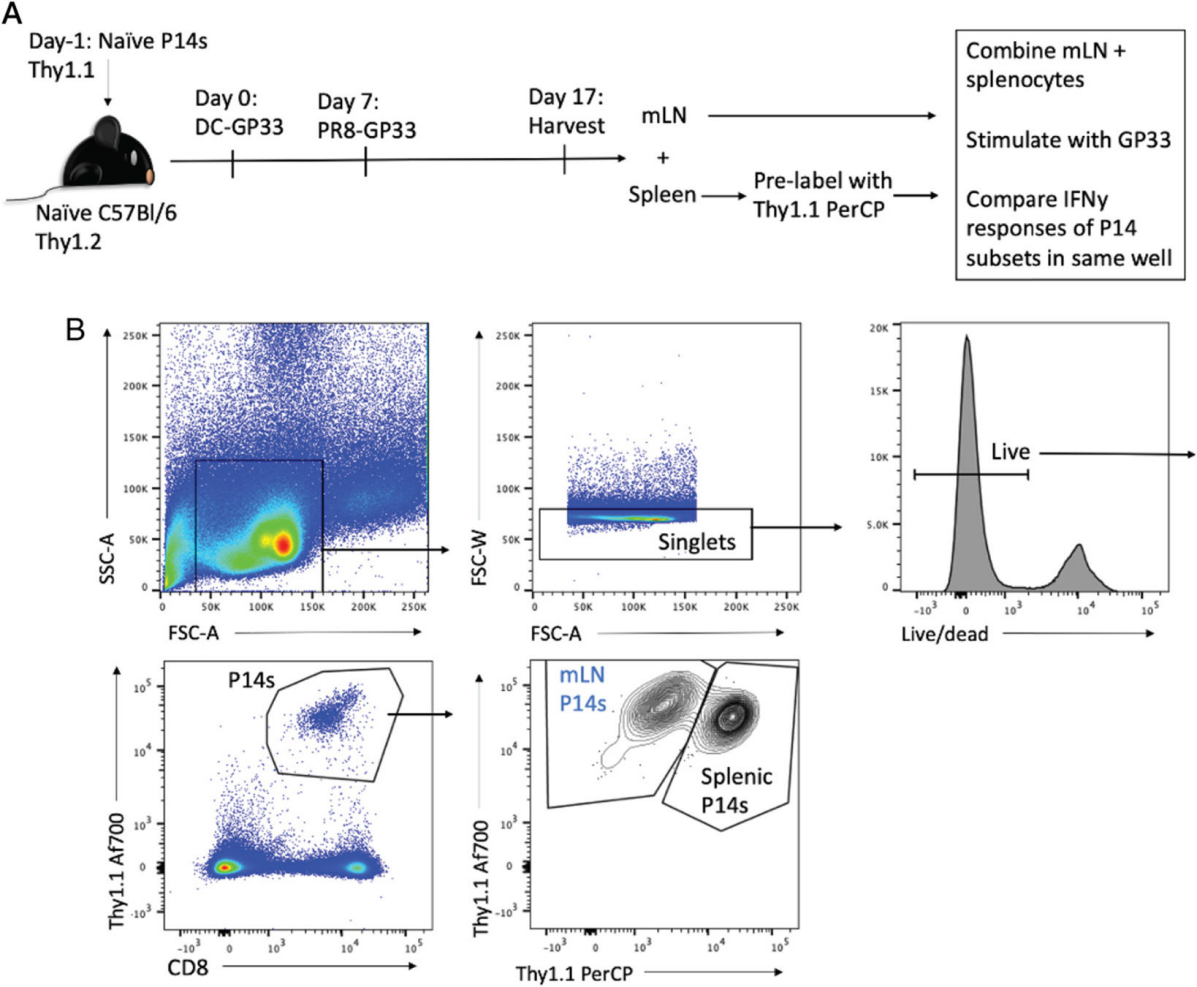
Experimental layout and gating strategy. (**A**) Experimental layout for comparing functional avidity of mLN P14 cells with splenic P14 cells after DC-gp33 prime and PR8-gp33 infection. (**B**) Gating strategy for identifying live P14 cells and differentiating mLN P14 cells from prelabeled splenic P14 cells after peptide stimulation.

For effector time points, lung draining mLNs and spleens were harvested 10 dpi, mononuclear cells isolated, and P14 cells stimulated by addition of titrated concentrations of the gp33 peptide. To differentiate two P14 populations in the same well, we prestained splenocytes with anti–Thy1.1-PerCP Cy5.5 before mixing with lymph node cells and in vitro peptide stimulation (as demonstrated in Fig. 1A). After stimulation, all cells were stained with anti–Thy1.1-Af700. This allowed identification of the entire Af700^+^ P14 population, then differentiation of PerCP Cy5.5-high splenic P14 cells from P14 cells derived from the mLN (Fig. 1B). This approach allowed a direct comparison of the IFN-γ production and functional avidity between these populations within the same APC environment, enabling a comparison of CD8 T cell–intrinsic phenotypes. It should be noted that among spleen cells, only P14 cells were stained with anti-Thy1.1 (Fig. 1) and not the remainder of the spleen cells that serve as APC populations.

### Effector mLN P14 cells exhibit increased functional avidity relative to splenic P14 cells

During a CD8 T cell response to an infection, activated CD8 T cells undergo a rapid expansion in numbers, giving rise to an effector population that acquires functions such as inflammatory cytokine production to combat the infection. After peak expansion, 90–95% of cells die via apoptosis, while the surviving cells go on to establish a memory population capable of rapidly responding and providing protection on reinfection (37). In the context of influenza infection, CD8 T cell responses peak in their magnitude in the lungs around 10 dpi (11). Before examining functional avidity at memory time points, we aimed to establish whether mLN P14 cells differed in their avidity relative to splenic P14 cells at an effector time point.

**FIGURE 2.**
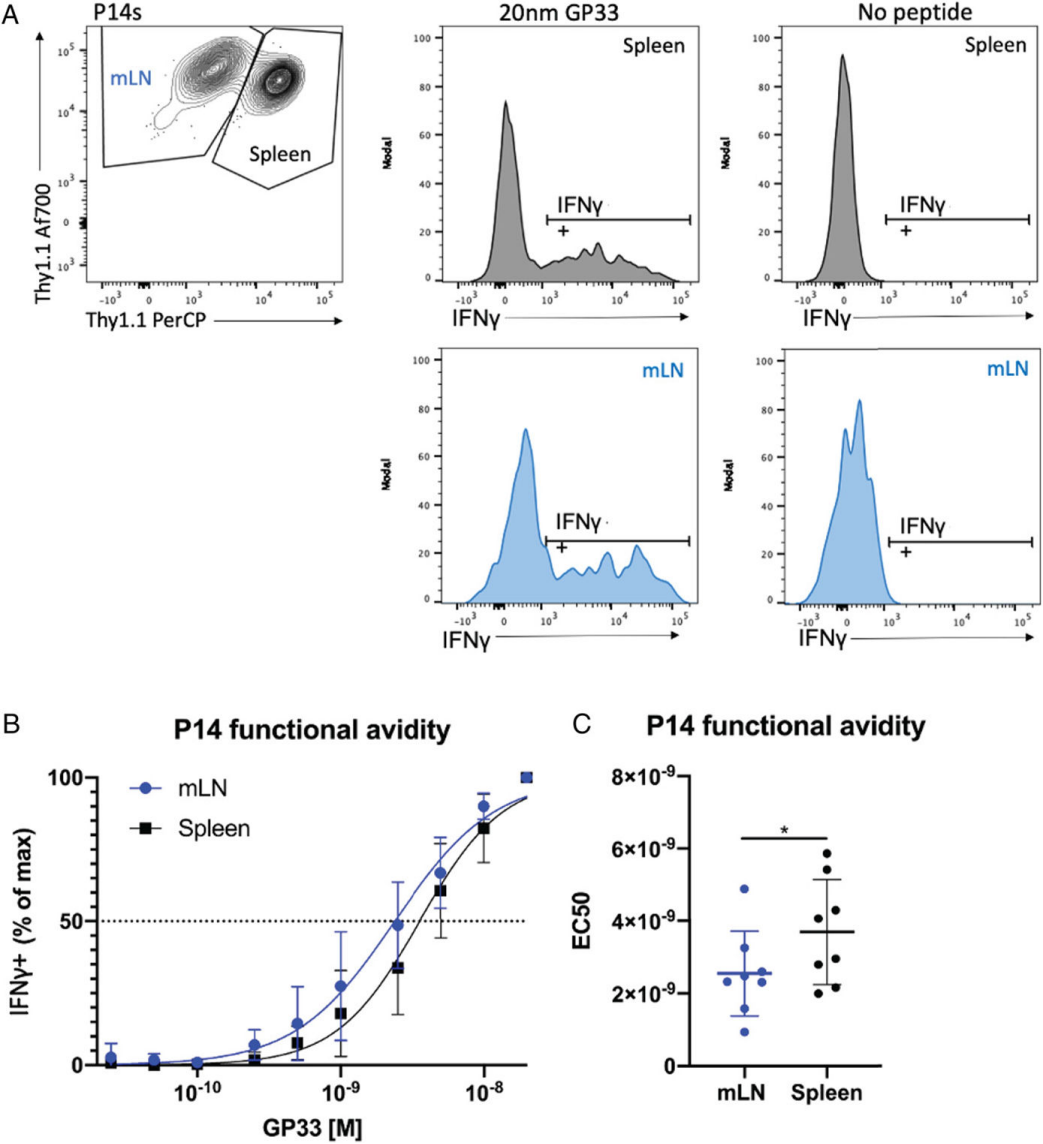
Functional avidity in mLN and splenic P14 cells 10 dpi with PR8-gp33 in DC-gp33-primed mice. P14 cells were isolated and identified as described in Fig. 1. (**A**) Gating on mLN and splenic P14 cells and their representative IFN-γ staining at the maximal peptide concentration (20 nM) and unstimulated control. (**B**) Functional avidity curves for mLN and splenic P14 cells, showing percentage of the maximal IFN-γ production across peptide concentrations. (**C**) EC_50_ values calculated from avidity curves in (B). *p < 0.05, analyzed via Student’s paired *t* test, with pairing of P14 cells from the same mouse (stimulated within the same well). (B) Mean ± SDs for each subset at each peptide dilution are shown. (C) Each dot represents a single mouse; means ± SDs are shown. Pooled data (*n* = 8) are reflective of two independent experiments (*n* = 4).

We found that mLN P14 cells exhibited a discernably enhanced functional avidity relative to splenic P14 cells, quantified by a decrease of ~1.5-fold in EC_50_ (Fig. 2B, 2C). This could reflect increased functional avidity in effector CD8 T cells responding to IAV Ag in the mLN or loss of functional avidity of effector CD8 T cells in the spleen.

### CD103^+^ mLN Trms exhibit increased functional avidity at memory time points

**FIGURE 3.**
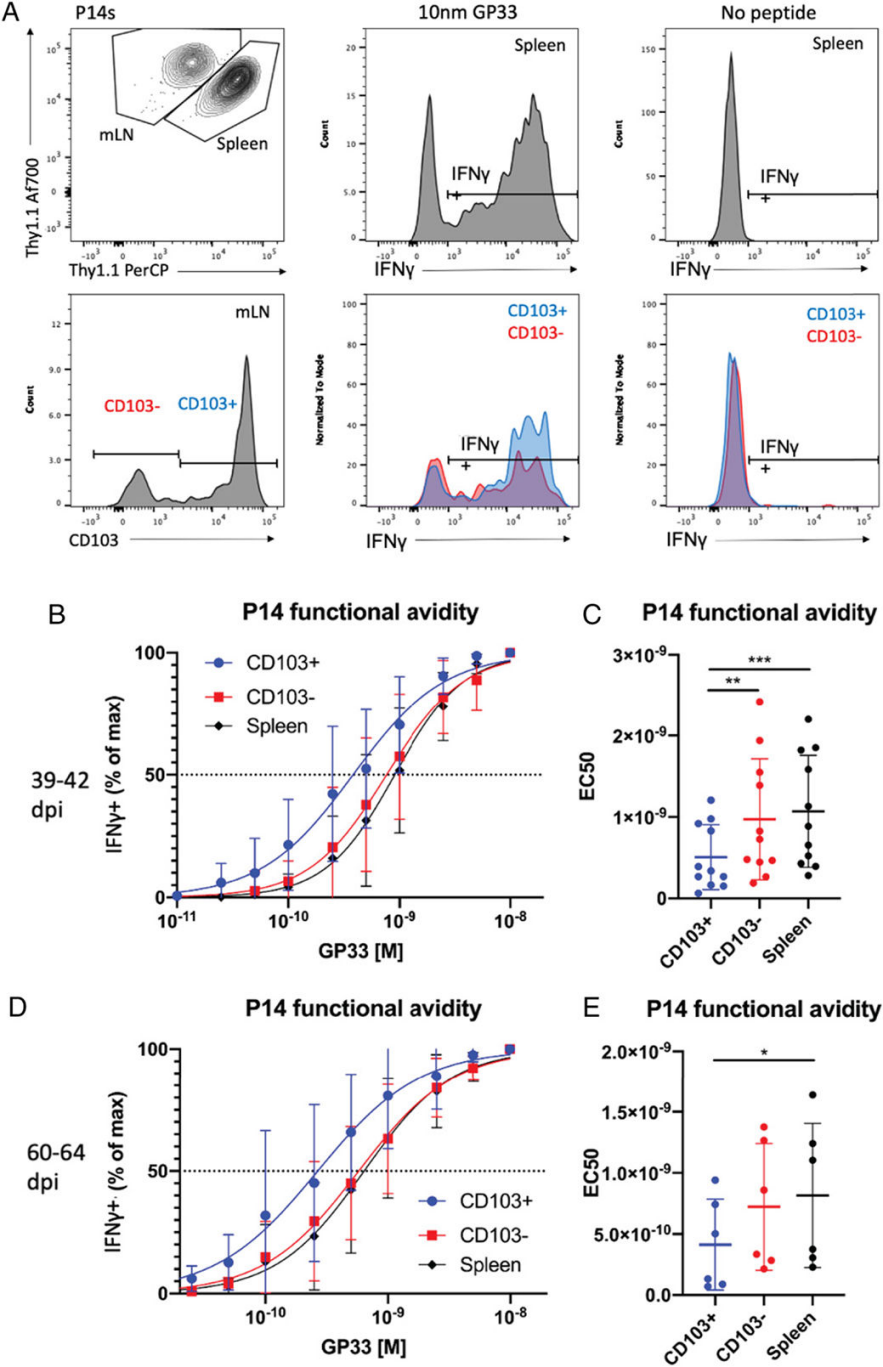
Functional avidity in mLN and splenic P14 cells at memory time points with PR8-gp33 in DC-gp33–primed mice. P14 cells were isolated and identified as described in Fig. 1. (**A**) Gating on mLN and splenic P14 cells and their representative IFN-γ staining at the maximal peptide concentration (10 nM) and unstimulated control. (**B**) Functional avidity curves for mLN and splenic P14 cells, showing percentage of the maximal IFN-γ production across peptide concentrations 39–42 dpi. (**C**) EC_50_ values calculated from avidity curves in (B) 39–42 dpi. (**D** and **E**) The same analyses as (B) and (C), except that pooled data are shown for 60–64 dpi. *p < 0.05, **p < 0.01, ***p < 0.001 analyzed via one-way ANOVA with Tukey’s multiple comparison test and matching of P14 cells from the same mouse (stimulated within the same well). (B and D) Mean ± SDs for each subset at each peptide dilution are shown. (C and E) Each dot represents a single mouse; means ± SDs are shown. (B and C) Pooled data (*n* = 11) are reflective of three independent experiments (*n* = 3–4). (D and E) Pooled data (*n* = 6) are reflective of two independent experiments (*n* = 3).

To assess functional avidity of IAV-induced memory CD8 T cells and the mLN Trm subset in particular, we harvested spleens and mLNs at 39–42 dpi. P14 cells were stimulated, identified, and differentiated as described earlier. Then, mLN Trms were identified through expression of CD103 (Fig. 3A), as we have recently demonstrated through parabiosis that influenza-induced CD103^+^ mLN CD8 T cells are tissue resident (38). We did not evaluate CD69, a conventional Trm marker, because its expression is upregulated by TCR stimulation (39). CD103^+^ mLN Trms were found to have a discernably increased functional avidity relative to both CD103^+^ mLN P14 cells and splenic P14 cells (Fig. 3B, 3C). These data suggested that CD103^+^ mLN Trms may be poised to respond rapidly on reinfection because of their high sensitivity to Ag.

To determine whether CD103^+^ mLN Trms remained more sensitive to Ag stimulation, we also assessed functional avidity at 60–64 dpi. Again, functional avidity was increased in CD103^+^ mLN Trms relative to splenic P14 cells (Fig. 3D, 3E). Although the difference in functional avidity between CD103^+^ and CD103^−^ mLN P14 cells was not significant at these time points (*p* = 0.0069), CD103^−^ mLN P14 cells were not found to be discernably more avid than splenic P14 cells. Together, these data indicate that the increased functional avidity seen in CD103^+^ mLN P14 cells may relate to the regulation of Trm phenotype rather than the mLN tissue environment as a whole.

### CD103^+^ mLN Trms express increased TCR

Although a number of factors can regulate functional avidity in CD8 T cell populations, the observation of increased avidity in CD103^+^, but not CD103^−^, memory P14 cells within the mLN suggested that this phenotype was regulated on a cell-intrinsic level, as opposed to arising from differences in the tissue environment such as APC function or the cytokine milieux. Using our costimulation technique, all Ag-specific CD8 T cell populations should encounter peptide–MHC (pMHC) complexes at the same surface density on APCs. However, the number of TCR–pMHC interactions a CD8 T cell receives could still be increased by an elevated surface TCR expression. Therefore, we investigated whether CD103^+^ mLN P14 cells might express a higher level of TCR to enable their enhanced functional avidity.

**FIGURE 4.**
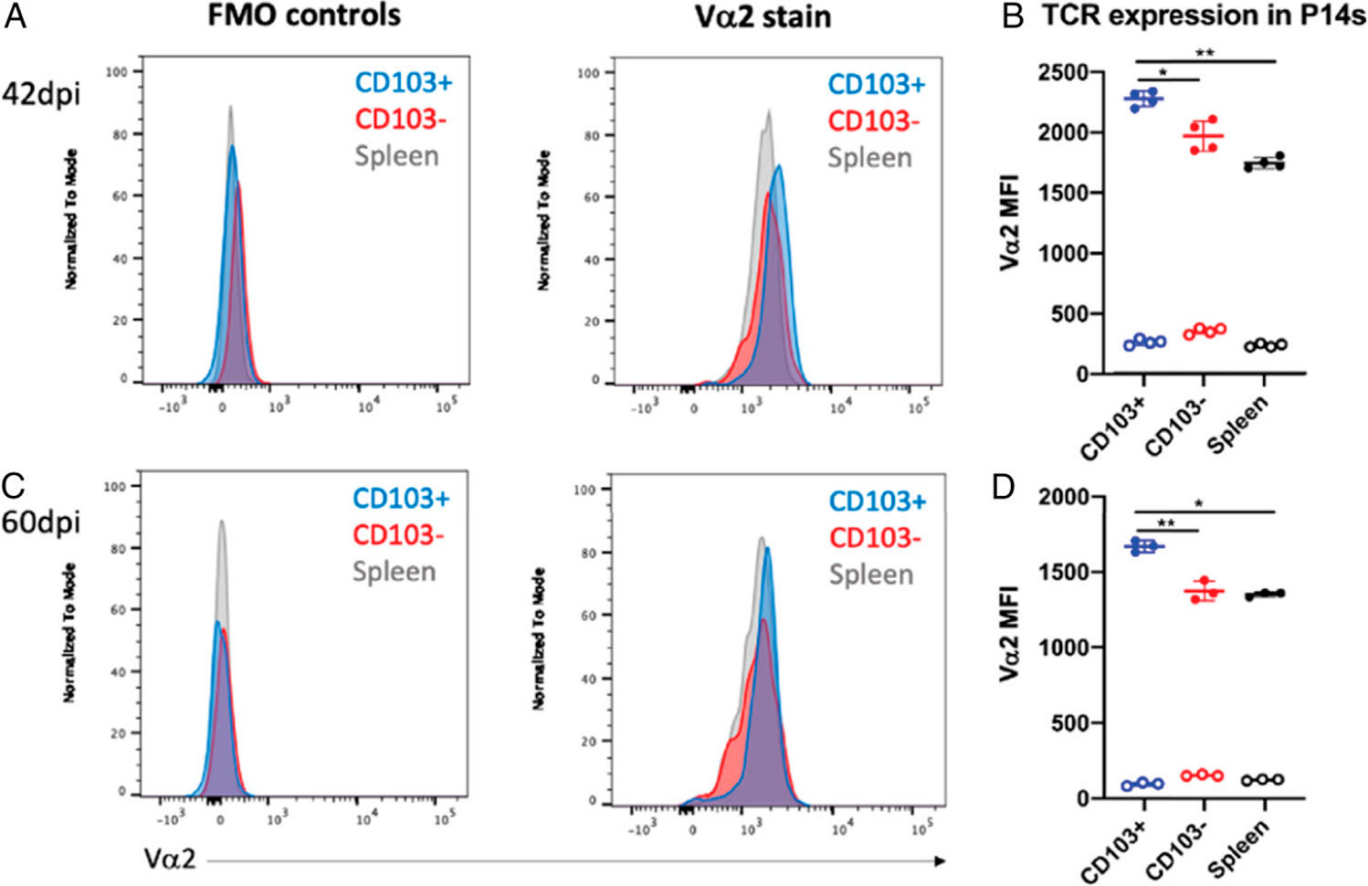
TCR expression in mLN and splenic P14 cells at memory time points with PR8-gp33 in DC-gp33–primed mice. mLN and splenic P14 cells were isolated after DC-gp33 prime and PR8-gp33 infection as described in previous experiments. (**A** and **C**) Representative Va2 staining and FMO controls for unstimulated mLN and splenic P14 cells at memory time points indicated to the left. (**B** and **D**) MFI of Va2 is shown for each subset. Ab-stained samples are shown in shaded circles, whereas FMO MFIs are shown in open circles. *p < 0.05, **p < 0.01, analyzed via one-way ANOVA with Tukey’s multiple comparison test and matching of P14 cells from the same mouse (stained within the same well). (B and D) Each dot represents a single mouse; means ± SDs are shown (*n* = 3–4).

We analyzed TCR expression in P14 cells from the mLN and spleen (obtained at 42 and 60 dpi in experiments shown in previous figures), combined them, and stained in the same well. Because P14 cells are TCR-Tg and express a fixed TCR, we assessed expression of the shared α-chain Vα2. We included a fluorescence minus one (FMO) control to detect any differences in autofluorescence or background signal caused by spectral overlap of other fluorophores used in the panel. Representative histograms demonstrate that there were no major differences in background fluorescence in the FMO, whereas differences in Vα2 staining can be observed in the wells containing the anti-Vα2 Ab (Fig. 4A, 4C). At an early memory time point of 42 dpi, CD103^+^ mLN Trms were found to express more TCR than either CD103^−^ mLN P14 cells or splenic P14 cells, as indicated by a higher median fluorescence intensity (MFI) of Vα2 (Fig. 4B).

Interestingly, this pattern in TCR expression corresponds with the observed pattern in functional avidity. The most functionally avid population, CD103^+^ mLN Trms, expressed the most TCRs. Likewise, the least functionally avid population, splenic P14 cells, expressed the least TCRs (Fig. 4B). This relationship was maintained at a later memory time point, with CD103^+^ mLN Trms expressing more TCR than CD103^−^ mLN P14 cells or splenic P14 cells at 60 dpi (Fig. 4D). Differences in TCR expression between these populations were not observed at an effector time point (data not shown). Together, these data indicate that CD103^+^ mLN Trms express more TCRs, which could serve as a mechanism for their enhanced functional avidity relative to mLN non-Trms or splenic memory CD8 T cells.

### i.n.^−^ i.v.^−^ CD103^+^ lung Trms do not exhibit enhanced functional avidity

**FIGURE 5.**
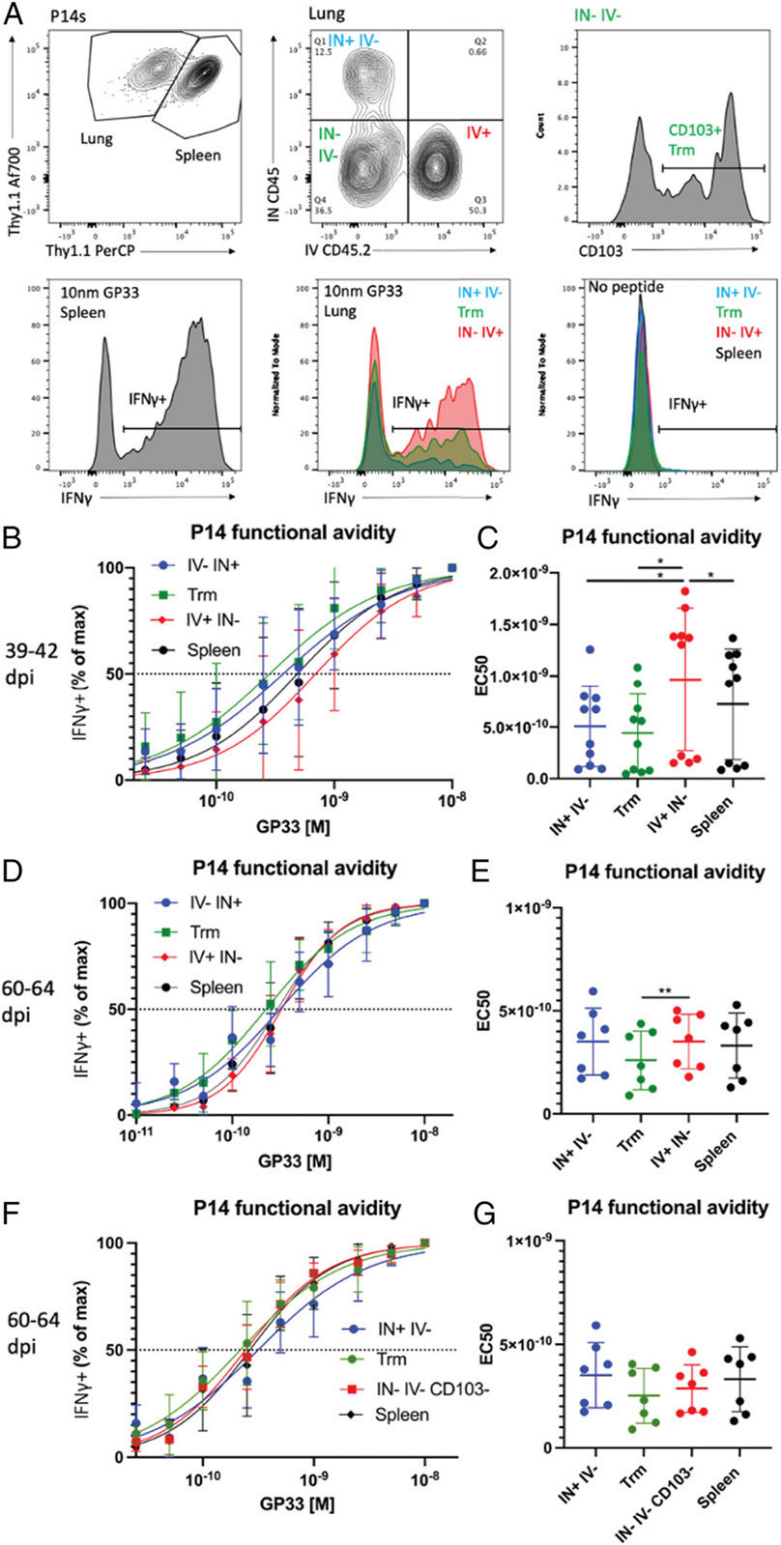
Functional avidity in lung and splenic P14 cells at memory time points with PR8-gp33 in DC-gp33–primed mice. P14 cells were isolated and identified as described in Fig. 1. (**A**) Gating on lung and splenic P14 cells and their representative IFN-γ staining at the maximal peptide concentration (10 nM) and unstimulated control. (**B**) Functional avidity curves for lung and splenic P14 cells, showing percentage of the maximal IFN-γ production across peptide concentrations 39–42 dpi. (**C**) EC_50_ values calculated from avidity curves in (C) 39–42 dpi. (**D** and **E**) The same analyses as (B) and (C), respectively, except that pooled data are shown for 60–64 dpi. (**F** and **G**) The same samples as (D) and (E), respectively, except that i.v.^−^ i.n.^−^ CD103^−^ P14 cells are shown instead of i.v.^+^ i.n.^−^ P14 cells. *p < 0.05, **p < 0.01, analyzed via one-way ANOVA with Tukey’s multiple comparison test and matching of P14 cells from the same mouse (stimulated within the same well). (B, D, and F) Means ± SDs for each subset at each peptide dilution are shown. (C, E, and G) Each dot represents a single mouse; means ± SDs are shown. (B and C) Pooled data. (F and G) The same analyses as (D) and (E), respectively, except that i.v.^−^ i.n.^−^ CD103^−^ P14 cells are shown instead of i.v.^+^ i.n.^−^ P14 cells. *p < 0.05, **p < 0.01, analyzed via one-way ANOVA with Tukey’s multiple comparison test and matching of P14 cells from the same mouse (stimulated within the same well). (B, D, and F) Mean ± SDs for each subset at each peptide dilution are shown. (C, E, and G) Each dot represents a single mouse; mean ± SDs are shown. (B and C) Pooled data (*n* = 10) are reflective of three independent experiments (*n* = 3–4). (D–G) Pooled data (*n* = 7) are reflective of two independent experiments (*n* = 3–4).

Given the role of lung Trms in providing heterosubtypic immunity against influenza, we aimed to determine whether lung Trms also exhibited an enhanced functional avidity relative to splenic P14. To label cells located in different compartments within the lung, we performed i.n. administration of anti-CD45 allophycocyanin to label P14 cells located in the airway of the lung (40). We also preformed i.v. injections to label cells in the vasculature through inoculation of anti-CD45.2 BV421, then harvested lungs and spleens. Using these labels in combination with our strategy for differentiating splenic P14 cells (Fig. 1A, 1B), we were able to identify i.v.^+^ lung P14 cells, i.v.^−^ i.n.^−^ CD103^+^ parenchymal Trm P14 cells, and i.n.^+^ i.v.^−^ airway P14 cells (Fig. 5A).

We did not observe an increase in functional avidity in airway or parenchymal Trm P14 cells relative to splenic P14 cells at 39–42 dpi (Fig. 5B, 5C). i.v.^+^ lung P14 cells exhibited a decreased functional avidity relative to all other populations (Fig. 5B, 5C), indicating that circulating memory CD8 T cells may be less sensitive to stimulation. At 60–64 dpi, decreased avidity in the i.v.^+^ compartment was only significant versus the parenchymal Trm population (Fig. 5D, 5E). At both memory time points, CD103^+^ parenchymal Trms did not exhibit increased functional avidity relative to splenic Trms (Fig. 5D, 5E). Given that CD103 could be used to differentiate mLN P14 cells of higher and lower avidity (Trms and non-Trms), we also compared CD103^+^ versus CD103^−^ P14 cells within the i.v.^−^ i.n.^−^ compartment of the lung at 60–64 dpi. We did not observe any significant difference in functional avidity between CD103^+^ and CD103^−^ i.v.^−^ i.n.^−^ P14 cells (Fig. 5F, 5G). This provides further evidence that enhanced functional avidity in CD103^+^ mLN P14 cells relative to CD103^−^ mLN P14 cells is a unique feature of mLN Trms, rather than a general feature of CD103^+^ CD8 T cells.

## DISCUSSION

In this study, we have established a methodology for comparing functional avidity across tissues, enabling the comparison of tissue-resident memory CD8 T cells with splenic CD8 T cells. Using this method, we have observed that functional avidity is dynamically regulated in the mLNs after influenza virus infection. At effector time points postinfection with PR8-gp33 after DC-gp33 prime, mLN P14 cells have an increased avidity relative to splenic P14 cells. At memory time points, CD103^+^ mLN P14 Trms have an increased functional avidity relative to splenic memory P14 cells (which are largely CD103^−^; data not shown). This phenotype was not observed in nonresident (38) CD103^−^ mLN P14 cells, suggesting that the sensitivity of the mLN Trm population is not merely environmentally driven and is instead part of the tissue residency phenotype within the mLN. Given that avidity is slightly enhanced in the mLN at effector time points, this raises the question whether Ag sensitivity plays a role in determining which cells differentiate into Trms in the mLN. Notably, we did not observe increased functional avidity of lung CD103^+^ Trms at the same memory time points. These data suggest that expression of CD103 itself is not the determining factor for the enhanced functional avidity exhibited by mLN Trms.

In the future, it would be of interest to determine whether enhanced functional avidity is a common phenotype of lymph node–resident CD8 T cell populations generated after other infections, beginning with additional models of respiratory infection. In addition to considering other pathogens, it would be of interest to examine functional avidity in CD8 T cells of different Ag specificities and in polyclonal endogenous populations. This could determine whether differences in TCR affinity in Trms integrate with the regulation of functional avidity to generate larger shifts in our CD8 T cell populations of interest (27, 32).

In addition to further unraveling the dynamics of enhanced avidity in CD103^+^ mLN Trms, it will be of interest to more deeply investigate the mechanisms underlying said avidity. In this article, we have demonstrated that surface TCR expression correlates with the functional avidity of memory P14 cells in the mLN and spleen after IAV infection. Although it is unsurprising that higher levels of TCR expression in mLN Trms would lead to increased signaling in response to a low density of pMHC complexes, this observation raises two major questions: first, how TCR upregulation occurs on these cells mechanistically; and second, whether mLN Trms possess other alterations to enable TCR signaling, such as enhanced expression of downstream signaling molecules.

The direct role of enhanced TCR expression and functional avidity in mLN Trms in controlling influenza virus on rechallenge is difficult to determine, given that the role of mLN Trms cannot be easily separated from that of lung Trms, which we did not observe to exhibit increased functional avidity. However, mLN Trm P14 cells generated through sequential infection with PR8-gp33 are capable of reducing mLN viral titers after rechallenge with LCMV, demonstrating that mLN Trms have the ability to control viral infection (38). The data presented in this article indicate that mLN Trms also have an enhanced functional avidity that could allow them to exhibit this viral control in response to lower levels of Ag presented at early stages of infection. Although the mLN is not a site of replication for seasonal influenza virus, mLN Trm populations could form a second line of defense against respiratory pathogens (38), because they are capable of retaining high sensitivity and functionality without the threat of inflammatory damage to the airways.

In addition to the potential involvement of Trms in viral control in the mLN, mLN Trm populations generated after influenza infection are capable of migrating to the lung and establishing lung Trm populations after reencounter of Ag in the mLN (41). Therefore, enhanced functional avidity could allow them to rapidly respond to lung-derived APCs in the mLN and relocalize to the site of infection. This would position mLN Trms as a reservoir of highly functional influenza-specific memory cells in the mLN, without the potential for lung pathology associated with Trms localized to the parenchyma or airway. Furthermore, lung Trms may relocalize to the mLN through retrograde migration, supporting the idea of the mLN as a safe place to retain Trms after respiratory infections (10). The higher functional avidity of mLN Trms suggests that this tissue may be a reservoir of highly sensitive memory CD8 T cells to combat subsequent infections in the lungs.

Overall, these data demonstrate that functional avidity may be uniquely regulated in Trm populations versus other memory subsets. In addition to influenza, Trms play a key role in immunity against many other infections wherein their avidity remains to be investigated. In this article, we have established a methodology to compare functional avidity across tissues while controlling for factors such as APC function. This could provide a new functional readout of use in characterizing Trm function after other infections or vaccinations. Achieving a greater understanding of the mechanisms underlying Trm responsiveness to Ag may allow the design of vaccines to optimize these functions and harness the ability of Trms to provide robust protection against pathogens such as influenza.

## Abbreviations used in this article:

Cat: catalog number
DC: dendritic cell
dpi: days postinfection
FMO: fluorescence minus one
HA: hemagglutinin
IAV: influenza A virus
i.n.: intranasal
LCMV: lymphocytic choriomeningitis virus
MFI: median fluorescence intensity
mLN: mediastinal lymph node
NA: neuraminidase
pMHC: peptide–MHC
Tg: transgenic
Trm: tissue-resident memory CD8 T cell
